# A pilot cluster randomised trial of the medicines and alcohol consultation (MAC): an intervention to discuss alcohol use in community pharmacy medicine review services

**DOI:** 10.1186/s12913-020-05797-z

**Published:** 2020-10-12

**Authors:** Duncan Stewart, Anne van Dongen, Michelle Watson, Laura Mandefield, Karl Atkin, Ranjita Dhital, Brent Foster, Brendan Gough, Catherine Hewitt, Mary Madden, Stephanie Morris, Ronan O’Carroll, Margaret Ogden, Steve Parrott, Judith Watson, Sue White, Cate Whittlesea, Jim McCambridge

**Affiliations:** 1 Department of Health Sciences, University of York, York, UK; 2 Department of Pharmacy, University of Reading, Reading, UK; 3 Whitworths Chemists Ltd, Scunthorpe, UK; 4 School of Social Sciences, Leeds Beckett University, Leeds, UK; 5 Department of Psychology, Stirling University, Stirling, UK; 6 North of England Commissioning Support (NECS), Newcastle, UK; 7 UCL School of Pharmacy, University College London, London, UK

**Keywords:** Alcohol, Community pharmacy, Medicine reviews, Pilot trial, Feasibility

## Abstract

**Background:**

Alcohol interventions are important to the developing public health role of community pharmacies. The Medicines and Alcohol Consultation (MAC) is a new intervention, co-produced with community pharmacists (CPs) and patients, which involves a CP practice development programme designed to integrate discussion of alcohol within existing NHS medicine review services. We conducted a pilot trial of the MAC and its delivery to investigate all study procedures to inform progression to a definitive trial.

**Methods:**

This cluster pilot RCT was conducted in 10 community pharmacies in Yorkshire, UK, with a CP from each who regularly conducted Medicine Use Review (MUR) and New Medicine Service (NMS) consultations. Randomisation was conducted using a secure remote randomisation service. Intervention CPs (*n* = 5) were trained to deliver the MAC in MUR/NMS consultations. Control CPs (n = 5) provided these services as usual. Consecutive MUR/NMS patients were asked by CPs to participate, screened for eligibility (consumption of alcohol at least twice per week), and baseline data collected for those eligible. A two-month follow-up telephone interview was conducted. Blinding of CPs was not possible, but patients were blinded to the alcohol focus of the trial. Primary outcomes were total weekly UK units (8 g of ethanol per unit) of alcohol consumption in the week prior to follow-up, and confidence in medications management. Trial procedures were assessed by recruitment, attrition, and follow-up rates.

**Results:**

260 patients were approached by CPs to take part in the trial, 68% (*n* = 178) were assessed for eligibility and 30% (*n* = 54) of these patients were eligible. Almost all eligible patients (*n* = 51; 94%) consented to participate, of whom 92% (*n* = 47) were followed-up at 2 months; alcohol consumption was lower in the intervention arm and confidence in medication management reduced slightly for both groups. Exploration of recall issues at follow-up showed a high level of agreement between a two-item quantity/frequency measure and 7-day guided recall of alcohol consumption.

**Conclusions:**

The pilot trial demonstrates the feasibility of implementing the MAC in community pharmacy and trial recruitment and data collection procedures. However, decommissioning of MURs means that it is not possible to conduct a definitive trial of the intervention in this service.

**Trial registration:**

ISRCTN57447996

## Background

Pharmacists and pharmacy staff are the third largest professional health workforce after nurses and doctors [1, 2]. Pharmacies in community settings in the UK now offer a range of services to the general public designed to promote and protect health, including medicine reviews, sexual health screening, smoking cessation and alcohol interventions [3]. The UK Department of Health and other national bodies have recommended that pharmacy-based alcohol interventions should be piloted and evaluated as part of the developing public health function of community pharmacies [4–7].

We have completed the only previous randomised controlled trial (RCT) (*n* = 407, follow-up 80%) of an alcohol intervention within the community pharmacy setting worldwide [8], apart from one small pilot trial (*n* = 69, 29% follow-up) that was published in the grey literature only [9]. Our previous trial found: community pharmacists to be very willing to participate in an effectiveness trial; the community pharmacy setting to be highly conductive to brief alcohol discussions for clinical and public health purposes when people were approached opportunistically, most of whom did not see their drinking as problematic or in need of intervention, even though they were drinking at hazardous or harmful levels [10]; low levels of alcohol-specific knowledge and variability in brief intervention skills among pharmacists. The RCT found no differences in outcomes amongst those who received a dedicated brief intervention delivered by pharmacists designed to help participants think about and reduce their drinking compared to those who did not [8]. This approach followed the format of brief interventions developed in primary care [11, 12], and we concluded that an entirely different approach to intervention design [13], more firmly rooted in community pharmacy practice itself, was needed.

Firstly, rather than asking pharmacists to take on an entirely new public health role, there is unexplored potential in optimising the contribution made to health and well-being within the core pharmaceutical role itself. This could be achieved by integrating attention to alcohol within existing pharmacy service delivery, as opposed to having dedicated and separate consultations for alcohol, such as we evaluated in the earlier trial [8]. This does not negate the need for training in consultation skills, as shown in the previous trial.

Second, understanding the reasons why people attend community pharmacies in the first instance may provide a basis for better targeting of interventions [12, 13]. Some pharmacy services may lend themselves better than others to a patient assessing the impact of alcohol on their health as the basis of considering behaviour change. It is likely that consultations routinely made to discuss medicine use may provide valuable opportunities to consider the possible consequences of alcohol for the effectiveness of medications, and on health more generally.

This pilot trial was part of a 5-year programme that aims to co-produce with the pharmacy profession and with patients, and evaluate in a definitive cluster RCT, an intervention discussing alcohol within routine medication consultations [14–19]. The new intervention, the Medicines and Alcohol Consultation (MAC), co-produced with pharmacists and patients [15], was designed to be incorporated into existing National Health Service (NHS) services delivered by community pharmacists. Medicines Use Reviews (MURs) and the New Medicine Service (NMS) aim to improve patients’ understanding and use of their medications, with the latter focused on newly prescribed medication and both targeting medications for specific long-term conditions. The aims and content of these services are described in detail elsewhere [18].

The aim of this external pilot trial was to investigate all study procedures to inform progression to the definitive trial. Specific objectives addressed in this paper were to investigate the following trial procedures: the feasibility of the recruitment strategy for CP and patient participants; attrition from the trial during recruitment and at 2 month follow-up; delivery of the MAC practice development programme for intervention CPs; measurement of the proposed trial primary outcomes, including data quality issues associated with alcohol consumption recall bias.

## Method

### Trial design

This was a multi-site, cluster randomised controlled pilot trial with a 1:1 allocation ratio, with a nested participant-centred process evaluation. The trial ran from April to October 2019. Pharmacists in community pharmacies allocated to the intervention delivered the MAC consultation with patients in MUR and NMS reviews, after completing a practice development programme (see below). Those from pharmacies randomised to the control condition continued to provide the MUR and NMS as usual, and recruit participants to the pilot trial in the same manner as the intervention condition. In both cases the MUR was the primary service promoted for recruitment, and the CPs were invited to explore whether it was also possible to recruit and deliver the MAC intervention via the NMS.

The pilot trial received NHS research ethics approval (REC reference19/SW/0082) and is registered with the ISRCTN registry (ISRCTN57447996). Findings are reported according to the Consolidated Standards of Reporting Trials (CONSORT) guidelines for pilot and feasibility studies [20].

### Participants

#### Community pharmacists

Community pharmacies (*n* = 10) within one defined geographic area (within 1.5 h of travel time from York, UK) were recruited prior to randomisation. One CP from each pharmacy was eligible for the trial, excluding locums, trainees, and other temporary practitioners. Briefly, after initial advertising for expressions of interest in the trial, CPs were deemed eligible if they: conducted MURs; were interested in the opportunity for practice development; could attend intervention and research training days; and confirmed that there was no planned disruption in the pharmacy. Eligible CPs were then selected on the basis of: agreeing to approach approximately 30 patients to recruit a target of 10; having managerial approval to participate; being able to attend training on specific days; willingness to be randomized; and for consultations to be audio recorded (with patient consent).

#### Patients

Consecutive patients recruited to the MUR/NMS as usual were asked by CPs (in the pharmacy private consultation room) if they would be interested in taking part in a study about how pharmacists discuss patients’ health and wellbeing in medicines reviews. If patients accepted, the CP then asked if the patient would be willing to complete a brief screening form. The form included a single item alcohol screening question embedded in a range of other health and service utilisation questions: “how often do you have a drink containing alcohol?”. Patients were thus unaware of the alcohol study focus (see blinding below). Five response categories range from ‘never’ to ‘four or more times per week’. Patients were eligible if they consumed alcohol at least twice per week (in addition to being aged 18 and over and eligible for an MUR or NMS consultation). Patients were not eligible if they had received treatment for alcohol in the past 12 months. Eligible patients were provided with a study information statement and completed an informed consent form.

### Intervention

The purpose of the MAC is to integrate attention to alcohol within existing pharmacist-led medicine review services. It is designed to enhance CPs’ person-centred consultation skills, such that alcohol consumption can be raised with patients during medicine review consultations in connection with medications and the conditions for which these are being taken. Underpinning the delivery of the MAC is a 6-week practice development programme to equip CPs to support patients to discuss and make informed decisions about their alcohol and medication use. Thus, the intervention comprised both the MAC practice development programme and the delivery of the MAC by participating CPs. The MAC programme comprised the following components:

1. Two practice development training days. The first day focused on core person-centred consultation skills (e.g. asking open questions), using the MAC in consultations, and preparing a practice development plan. The second was scheduled 3 weeks later and focused on the key issues identified in using the MAC in practice and included more advanced person-centred skills and case studies.
2. A four-page paper-based MAC guide summarising the structure of the MAC and core content within consultations. The MAC guide provided six steps within which the CP could flexibly organise the medicine review consultation to be responsive to patient agendas and explore possible connections between alcohol consumption, use of medicines and the patients’ health. It was introduced to CPs in the first training day.
3. A range of learning support resources, including case studies, information about interplay between alcohol and specific medications, and practice development exercises were offered at each training day, with audio-recording of consultations introduced on the second training day (see below).
4. Individually tailored weekly practice development support site visits or telephone calls by the MAC support team, delivered for the 3 weeks between training days 1 and 2, and for a further 3 weeks after training day 2 (before patient recruitment). Audio recording of consultations (with patient consent) were used to facilitate discussions of practice development and use of person-centred consultation skills and the MAC.
5. Invitation to engage in peer support (buddying in pairs and group discussions over WhatsApp).

### Follow-up procedures

Contact details and preferences for consenting participants were collected by the trial CPs. A trained researcher collected outcome data by telephone from participants 2 months after recruitment to the study. Participants were contacted by telephone at least three times to arrange the follow-up interview, and if unsuccessful a self-completion questionnaire was posted with a stamped addressed envelope for return.

### Outcomes

Trial procedural outcomes were: recruitment of CPs; delivery of the MAC practice development programme; the proportion of patients approached for the trial who accepted the initial invitation; the proportion of patients accepting the invitation who were eligible for the trial; the proportion of eligible patients who consented; and the proportion of recruited participants who provided follow-up data (interview or postal questionnaire). Candidate primary outcome measures for the main trial were total weekly UK units (8 g of ethanol per unit) of alcohol consumption in the 7 days prior to follow-up; and confidence in medications management measured using the PROMIS Self-Efficacy for Managing Medications and Treatment scale (6 item version) [21]. Candidate secondary clinical outcomes were: quality of life measured by the EQ. 5D-5L [22]; adherence measured by ProMAS [23]; anxiety (GAD-7) [24] and depression (PHQ-8) [25].

### Sample size

At least four clusters per arm are recommended for cluster pilot randomised, controlled trials [26]. Assuming an average of 8 participants per pharmacy are recruited, we planned to recruit 80 participants from 10 pharmacies (equivalent to 70 participants in an individually randomised trial, assuming intraclass correlation coefficient (ICC) = 0.02). Based on earlier (unpublished) feasibility work, we estimated that approximately a third of MUR patients would be eligible for the trial, and each CP was asked to approach at least 30 patients in the planned 8 weeks of patient recruitment. A trial of this size allows a completion rate of 80% to be estimated within a 95% confidence interval of ±9% and participation rate of 50% within ±8% [27].

### Randomisation and blinding

Randomisation of pharmacies was undertaken by an independent statistician using minimisation (taking account of urban vs rural setting, independents vs multiples, and above and below median Index of Multiple Deprivation score). Minimisation was undertaken in minimPy using naïve minimisation with base probability 1.0 (i.e. deterministic minimisation) using marginal balance as the distance measure and with minimisation factors having a weighting of 1. Randomisation was at the level of the CP. CPs randomised to the control continued to provide the MUR and NMS as usual. Those randomised to the intervention were exposed to the MAC programme and used the MAC guide in consultations. By the nature of the intervention, blinding of the CPs was not possible. Participants were blinded to the alcohol focus of the trial in order to safeguard the unbiased evaluation of highlighting alcohol in the MUR/NMS consultations as alcohol assessment reactivity is a well-established phenomenon in trials [28]. The study was described to potential participants by CPs as a study to help improve medicines reviews.

### Analysis

All analyses were conducted in R [29] following the principles of intention-to-treat with participant outcomes analysed according to their original, randomised group, where data are available, irrespective of deviations based on non-compliance. As this was a pilot trial, outcomes were intended to inform planning and delivery of a definitive trial only and thus the trial was not powered to detect any intervention effect. Given these objectives, we did not include those lost to follow-up in the analyses of outcomes. For the primary clinical outcomes, mean differences and their 95% confidence intervals were calculated using a mixed effects model including pharmacy as a random effect and baseline measure and treatment as fixed effects. No methods of imputation were utilised to explore the robustness of findings to missing data. Following developer’s guidance [30], the PROMIS Self-Efficacy for Managing Medications and Treatment scale raw scores (scaled up to 8 items) were converted to t-scores. For all clinical outcomes, mean, standard deviation, median and interquartile range (IQR) are presented by treatment condition.

An exploratory sub-analysis was conducted for the measurement of alcohol consumption at follow-up. The trial design included an assessment of whether two single item alcohol frequency (number of drinking days in the past 7 days) and quantity (units of alcohol consumed on a typical drinking day, in the past 7 days) measures performed as well as guided retrospective 7 day drinking recall. The retrospective measure asked participants to start with the previous day, and to recall if they drank any alcohol on this day. If they responded “yes”, questions were asked to establish the type, brand or strength and quantity of each drink consumed. This procedure was repeated for each of the last 7 days, and the information converted into units of alcohol (where one unit approximates to 8 g of ethanol). Patients were randomly allocated to a follow-up including both measurement approaches, or the frequency/quantity measure only. Agreement between the two measures was assessed ICCs with 95% confidence intervals. Participants were also randomised to a one or 2 month recall period for primary care service utilisation questions (likely to be the most frequent health service contact for this patient group) to assess potential recall issues (data not reported).

## Results

### Community pharmacist and patient recruitment

**Fig. 1:**
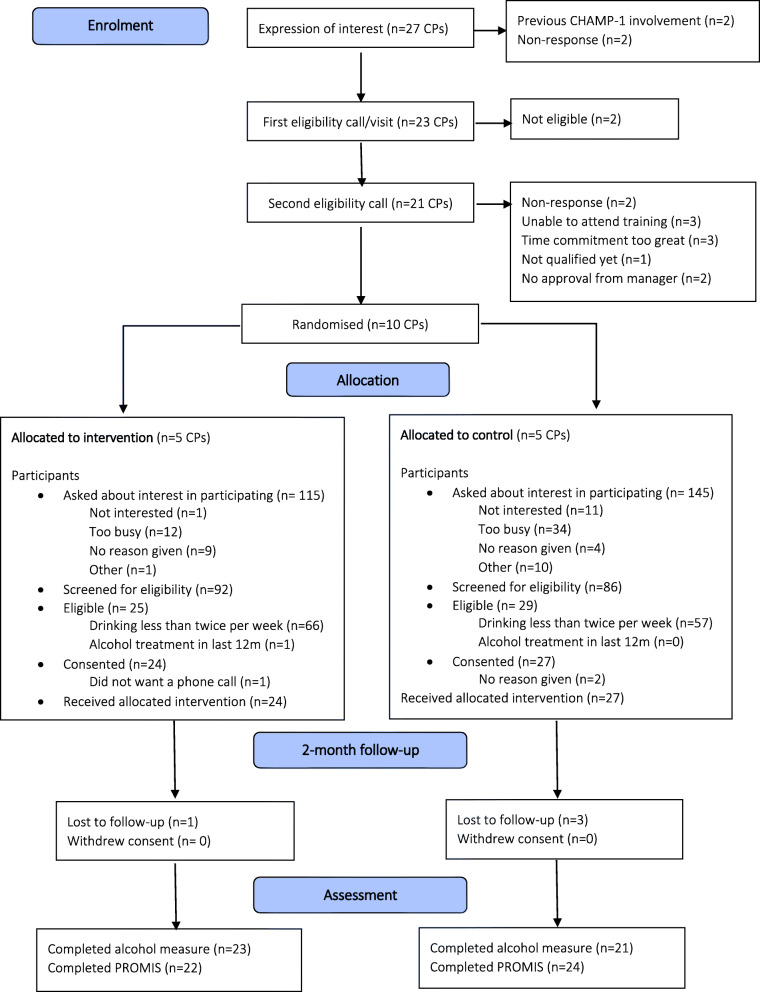
Pilot trial flow diagram

The flow of participant recruitment and retention is shown in Fig. 1, with data for individual CPs shown in Table 1. There were 27 CPs who expressed interest in the trial and 10 were randomised (5 in each arm). All 5 intervention CPs completed the MAC programme, before patient recruitment commenced. Overall, 260 patients were approached by CPs to take part in the trial during 12 weeks of recruitment, of whom 68% (*n* = 178; range 19 to 100% per CP) agreed to be screened for eligibility. A higher number of patients were approached in the control arm than in the intervention arm (145 versus 115), but a higher proportion of intervention arm patients than control arm patients approached accepted the invitation to take part (80% (*n* = 92) versus 59% (*n* = 86)). Thirty percent (*n* = 54; range 12 to 57% per CP) of patients screened were eligible for the trial (i.e. drank alcohol twice per week or more). Almost all eligible patients consented to take part in the trial (96%; 2 refused and 1 did not complete contact details for follow-up). All but 6 eligible patients were recruited via the MUR. A total of 51 patients consented. The median cluster size was four in the intervention arm and five in the control arm. Participant baseline demographic and clinical characteristics are summarised in Table 2.

**Table 1:** Patient recruitment and retention per pharmacy

Site	Trial arm	Approached	Initial invitation accepted		Eligible		Recruited	MUR	NMS	Followed- up
		n	n	%^a^	n	%^b^	n	n	n	n
1	Intervention	22	22	100	4	18	4	4	0	4
2	Intervention	11	9	82	4	44	4	4	0	4
3	Intervention	16	7	44	2	29	2	2	0	2
4	Intervention	37	37	100	8	22	8	5	3	7
5	Intervention	29	17	59	7	41	6	5	1	6
6	Control	34	17	50	2	12	2	2	0	2
7	Control	31	26	84	6	23	6	6	0	4
8	Control	31	6	19	3	50	1	1	0	1
9	Control	23	14	61	5	36	5	5	0	5
10	Control	26	23	88	13	57	13	11	2	12
**Total**		**260**	**178**	**68**	**54**	**30**	**51**	**45**	**6**	**47**

Note: ^a^Proportion of patients approached who accepted the initial invitation; ^b^Proportion of patients accepting the invitation who were eligible for the trial

**Table 2:** Baseline demographic and health related measures

	MAC (*N* = 24)	Usual Care (*N* = 27)	Total (*N* = 51)
**Sex**			
Female	9 (38%)	14 (52%)	23 (45%)
Male	15 (62%)	13 (48%)	28 (55%)
**Age (years)**			
Mean (SD)	70.71 (8.51)	62.78 (10.90)	66.51 (10.54)
Median (Q1, Q3)	72.50 (64.00, 75.00)	61.00 (53.00, 73.50)	67.00 (60.00, 74.00)
Min - Max	53.00–92.00	47.00–83.00	47.00–92.00
**Frequency of alcohol consumption**			
2 to 3 times per week	17 (71%)	17 (63%)	34 (67%)
4 or more times per week	7 (29%)	10 (37%)	17 (33%)
**Number of prescribed medications**			
Mean (SD)	4.54 (2.00)	4.58 (1.96)	4.56 (1.96)
Median (Q1, Q3)	4.00 (3.75, 6.00)	4.00 (3.25, 6.00)	4.00 (3.25, 6.00)
Min - Max	1.00–9.00	2.00–9.00	1.00–9.00
**EQ 5D-5L score**			
Mean (SD)	0.85 (0.17)	0.75 (0.24)	0.80 (0.22)
Median (Q1, Q3)	0.89 (0.72, 1.00)	0.84 (0.69, 0.86)	0.84 (0.70, 1.00)
Min - Max	0.46–1.00	−0.10 - 1.00	−0.10 - 1.00
**PROMIS raw score**			
Mean (SD)	26.75 (3.79)	28.41 (2.06)	27.63 (3.09)
Median (Q1, Q3)	28.00 (25.75, 30.00)	29.00 (27.50, 30.00)	29.00 (26.00, 30.00)
Min - Max	15.00–30.00	24.00–30.00	15.00–30.00
**PROMIS t-score**			
Mean (SD)	50.51 (8.34)	54.60 (6.87)	52.67 (7.80)
Median (Q1, Q3)	49.91 (45.92, 60.74)	54.95 (49.05, 60.74)	54.95 (46.56, 60.74)
Min - Max	32.76–60.74	42.89–60.74	32.76–60.74

### Attrition

No participants withdrew consent during the course of the study. Four participants (8%) were lost to follow up and did not complete the 2-month follow-up questionnaire (one in the intervention arm, three in the control arm). A total of 44 (86%) (23 intervention, 21 control) completed the primary alcohol measure at 2 months and 46 (90%) (22 intervention, 24 control) completed the PROMIS. Thirty-eight (81%) completed the follow-up data collection over the telephone and 9 (19%) completed a paper questionnaire and returned it via post.

### Primary clinical outcomes

The mean number of weekly alcohol units at 2 months was lower in the intervention arm (10.4, SD = 9.4) than in the control arm (14.2, SD = 13.8), although there was a higher variation in the responses in the control arm (Table 3). The adjusted (for pharmacy and baseline measure) mean difference was − 7.23 units (95% CI: − 17.87 to 2.99). PROMIS t-scores were lower in the intervention arm (48.8, SD = 7.5) than in the control arm (53.4, SD = 7.9). The adjusted mean difference was − 2.48 (95% CI: − 6.49 to 1.55).

**Table 3:** Outcomes at 2 months by treatment condition

Outcome measure	MAC			Usual Care			
	n	Median (IQR)	Mean (SD)	n	Median (IQR)	Mean (SD)	Adjusted mean difference (95% CI)
**Alcohol (Weekly units)**	23	8(4,16)	10.4 (9.4)	21	8 (3,24)	14.2 (13.8)	−7.23(−17.87, 2.99)
**PROMIS t-score**	22	46.6 (42.89,54.95)	48.8 (7.5)	24	53.5 (47.38,60.74)	53.4 (7.9)	−2.48 (−6.49, 1.55)
**PROMIS raw score**	22	26 (24,29)	26.2 (2.8)	24	28.9 (26.5,30)	27.8 (3.1)	
**ProMAS score**	22	13 (11,15)	13 (3)	23	13 (11,14)	12.3 (2.4)	
**PHQ-8 score**	23	1 (0,3)	2.2 (2.5)	24	1.5 (0,4)	2.2 (2.4)	
**GAD-7 score**	23	0 (0,3)	1.9 (2.9)	24	0 (0,1.5)	0.9 (1.7)	
**EQ 5D-5L score**	23	0.8 (0.8,1)	0.9 (0.1)	24	1 (0.72,1)	0.8 (0.2)	

### Secondary clinical outcomes

PHQ-8 and GAD-7 scores at 2 months were generally very low, indicating low levels of depression and anxiety in the recruited participants (Table 3). EQ. 5D-5L scores at 2 months were similar across treatment arms, with both arms having average scores close to 1 indicating little health-related impairment of quality of life. ProMAS scores for adherence were also similar in both arms, indicating medium to high medication adherence.

### Alcohol consumption measures sub-analysis

Comparison of total weekly alcohol units calculated from the quantity/frequency items and the guided retrospective 7 day drinking recall was conducted for a sub-sample of patients randomly allocated to both measures (*n* = 25). Of these, complete data were available for 19 patients and showed a high level of agreement: ICC = 0.91 (95% CI: 0.85 to 0.98). There was 100% agreement for frequency of consumption, and for quantity the ICC was 0.96 (95% CI: 0.93 to 0.99). In the majority of cases (12/19) the differences between the two alcohol measures were zero. Five had small differences (between 0.5 and 2 units), and for two patients the difference was exactly 14 units with higher estimates of consumption for the short quantity/frequency items in comparison to the guided retrospective 7 day drinking recall.

## Discussion

The findings from this pilot trial demonstrate the feasibility of conducting a definitive trial of this intervention in key respects: the MAC was acceptable to CPs and to patients and the MAC programme was implemented successfully; data collection procedures at baseline and follow-up were implemented as planned; a high proportion of eligible patients consented and were recruited to the trial; and attrition was very low. These findings compare favourably to the previous community pharmacy trial (98% of eligible patients consented; 80% follow-up rate at 3 months) [8], and the only previous UK medicine review RCT (85% follow-up rate at 10 weeks) [31]. The latter trial was not able to record the number of patients approached who declined to take part. It is, therefore, not possible to calculate a comparable proportion of eligible patients providing consent.

We made considerable efforts to support CPs conduct the trial, but there were marked variations between sites in the flow and recruitment of eligible patients. The majority of issues affecting patient recruitment (e.g. number of patients eligible for an MUR or NMS, number of patients agreeing to take part in an MUR or NMS) were outside of the control of the research team. The number of MUR or NMS patients approached to take part in the study was lower than anticipated and we did not meet the recruitment target, even after extending the recruitment period. By design, the target for the pilot was more demanding than for the planned definitive trial (5 vs 3 patients per CP per month). The timing of the study must be considered within the context of an exceptional period of uncertainty in community pharmacy, especially in terms of delivery of enhanced NHS services such as the MUR and NMS. Just before the start of the study, national changes to the NHS community pharmacy contractual framework introduced an intermediary reimbursable ceiling of 200 MURs for the months April–September, and in July, the annual limit was reduced to 250 for the financial year (it was 400 per year previously), to be followed by phasing out of the service in 2020/21 [32]. The vast majority of the pilot recruitment took place in MURs and this was the NHS service at the centre of our thinking for delivery of the MAC intervention. The changes impacted on pharmacy business plans, beyond the control of the participating pharmacists. For example, three participating pharmacies reached the revised MUR limit during the study. Discussions with the trial CPs indicated a re-orientation of priorities in their pharmacies, with MURs given less priority than other activities, and the much lower uptake of NMS was unable to make up the shortfall because it was seen as more challenging and/or less appropriate. The new community pharmacy contract means that proceeding with MURs is not feasible for a definitive trial and no longer of relevance to the NHS. We similarly concluded, after consulting with our practitioner and patient advisory groups, that the lack of fit with NMS consultations meant that it is also not feasible to conduct a trial of the MAC solely within the context of this service.

We took the opportunity to investigate data quality issues associated with alcohol consumption recall bias. The retrospective 7 day drinking measurement of consumption, using aided-recall techniques and allowing participants to describe the content and quantity of drinks consumed for each day, produces more valid consumption estimates than other approaches [33]. However, we were conscious of possible participant burden, especially in a telephone interview. The validity of brief two-item quantity/frequency measures for screening purposes is established [34], but we investigated the potential use of this approach for trial outcome measurement purposes. Although conducted with a small sub-sample, the findings showed a high level of agreement between the two item and 7 day drinking recall and it may be feasible to use the quantity/frequency measure as a primary outcome in a definitive trial if the large over-estimation by two participants is successfully addressed. Seven day recall of consumption provides higher estimates than longer recall periods [35], and the accuracy of recall deteriorates day by day [36], making alcohol consumption intrinsically difficult to measure without error. For example, the index week will not be representative of usual drinking behaviour for many.

Of the two candidate primary outcomes for the main trial, the findings for alcohol consumption were more indicative of change. However, the analyses were conducted to assess the appropriateness of the measures for a definitive trial only and should be treated with caution; the confidence intervals were wide due to the issues to do with the accuracy of the alcohol consumption measure and the small sample size.

## Conclusions

This pilot trial fulfilled the set aims and objectives and established within the context we were operating in the feasibility of undertaking a large trial in community pharmacies. The changes to the NHS mean that we will not proceed to conduct a definitive trial of the MAC in community pharmacy medicine review services, so the value of this study is in adding to the meagre evidence-base attesting to the feasibility of trials of medicine review services such as NMS [31]. In parallel to the decommissioning of MURs in community pharmacy, a new medicine review service is to be introduced in General Practice (GP), the Structured Medicine Review (SMR), alongside funding for a new GP pharmacist workforce to lead its delivery [37]. We are now in the position of adapting our original research plans, with the approval of our funder, to this new service setting. This pilot trial has provided evidence generating qualified confidence that a trial of the MAC delivered by pharmacists may be feasible within GP practices, returning to the setting in which brief interventions have been most extensively studied [13, 38], albeit with both a different practitioner group and approach to intervention development [39]. The MAC aligns well with the medicines optimisation focus of the new SMR [37] and the proposed enhanced role for pharmacists in prescribing and supporting patients with complex needs to better manage their medications. The pilot trial findings provide a solid foundation for adapting the intervention and the research to the primary care setting, with some further feasibility work needed before a definitive trial becomes possible.

## Data Availability

The data that support the findings of this study are available from the Principal Investigator (JM) upon reasonable request.

## Abbreviations

CP: Community Pharmacist
GP: General Practice
ICC: Intraclass Correlation Coefficient
MAC: Medicines and Alcohol Consultation
MUR: Medicines Use Review
NHS: National Health Service
NMS: New Medicine Service
RCT: Randomised Controlled Trial
SMR: Structured Medicine Review
